# Supplementary figures and images for: High-resolution density assessment assisted by deep learning of Dendrophyllia cornigera (Lamarck, 1816) and Phakellia ventilabrum (Linnaeus, 1767) in rocky circalittoral shelf of Bay of Biscay
Source: PeerJ. 2024 Mar 7;12:e17080. doi: 10.7717/peerj.17080 (PMC10924775; doi:10.7717/peerj.17080)

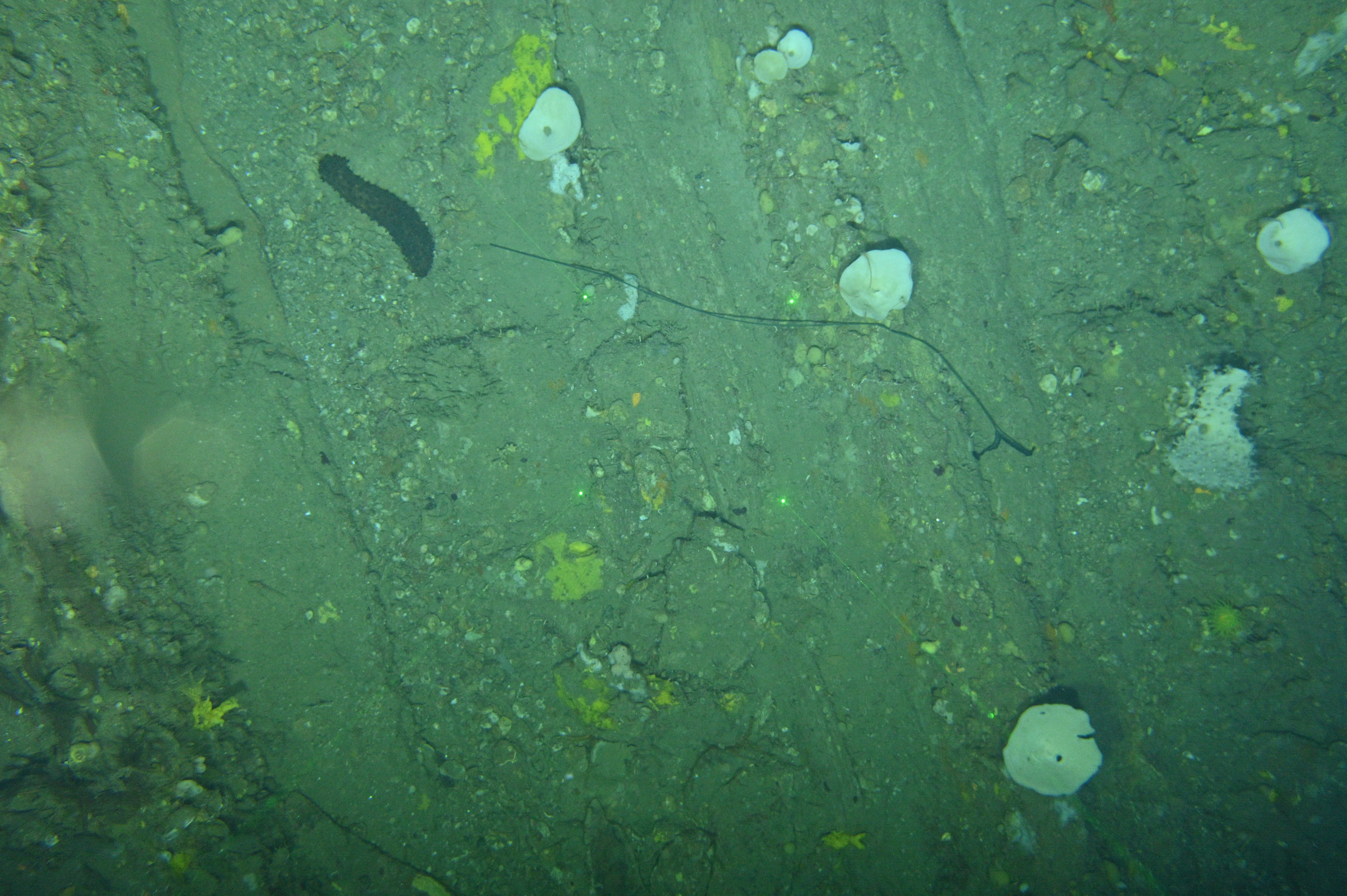

Supplement: Supplemental Information 6 [file peerj-12-17080-s006.jpg]
